# Laboratory monitoring of rivaroxaban in Chinese patients with deep venous thrombosis: a preliminary study

**DOI:** 10.1186/s40360-020-00414-5

**Published:** 2020-05-28

**Authors:** Ying Li, Liping Du, Xiaowan Tang, Yuexin Chen, Dan Mei

**Affiliations:** 1 Department of Pharmacy, Peking Union Medical College Hospital, Chinese Academy of Medical Sciences and Peking Union Medical College, Beijing, 100730 China; 2 Department of Pharmacy, National Cancer Center/Cancer Hospital, Chinese Academy of Medical Sciences and Peking Union Medical College, Beijing, 100021 China; 3 Department of Vascular Surgery, Peking Union Medical College Hospital, Chinese Academy of Medical Sciences and Peking Union Medical College, Beijing, 100730 China

**Keywords:** Rivaroxaban, UPLC-MS/MS, DVT, Concentration monitoring

## Abstract

**Background:**

Rivaroxaban, a novel oral anticoagulant drug, is widely used in clinical practice. There is no standardized laboratory monitoring for rivaroxaban, and its plasma concentration in Chinese patients with deep vein thrombosis is unclear. The rivaroxaban concentrations in human plasma and determine the steady-state concentration of rivaroxaban in patients with deep vein thrombosis are needed.

**Methods:**

An ultra-high-performance liquid chromatography with mass spectrometric detection method was developed. Chromatographic separation was performed on a Waters BEH C18 column with isocratic elution using a mobile phase composed of acetonitrile and water. Quantitation of the analytes was performed using positive ionization mode and mass transitions of m/z 437.3 → m/z 145.0 and m/z 440.1 → m/z 145.0 for rivaroxaban and the internal standard, respectively. Blood samples were collected at 0 h and 2 h after patients took rivaroxaban for 7 days or more.

**Results:**

The method was validated over the concentration range of 0.5 ~ 400 ng•mL^− 1^ with a very low limit of quantification of 0.5 ng·mL^− 1^, and the intra- and inter-day precision (RSD%) were < 15%. The range of the steady state concentration in patients that took 15 mg rivaroxaban twice daily, 10 mg twice daily, 20 mg once daily, 15 mg once daily, and 10 mg once daily were 168.5 ~ 280.1 ng•mL^− 1^, 74.2 ~ 271.4 ng•mL^− 1^, 25.7 ~ 306.8 ng•mL^− 1^, 24.5 ~ 306.4 ng•mL^− 1^, and 15.4 ~ 229.2 ng•mL^− 1^, respectively.

**Conclusions:**

The plasma rivaroxaban concentration in patients who took 10 mg rivaroxaban twice daily fluctuated less than that in patients who took 20 mg rivaroxaban once daily. The plasma concentration can be used for therapeutic drug monitoring for rivaroxaban.

## Background

Rivaroxaban is an oral anticoagulant that directly inhibits activated factor X (FXa) and is effective in the prevention of venous thromboembolism after orthopaedic surgery. Studies have demonstrated that the anticoagulant effect of rivaroxaban is similar to that of vitamin K antagonist (VKA), and there is no difference in the first major or clinically relevant nonmajor bleeding risk between rivaroxaban and VKA [1, 2]. Rivaroxaban can also reduce the major bleeding risk and increase the risk of gastrointestinal bleeding compared to vitamin K antagonist (VKA) [1]. Moreover, rivaroxaban has been reported to have predictable pharmacokinetics and pharmacodynamics [3, 4]. While routine monitoring is not required, there are many situations in which the need to assess the anticoagulant effect is required for clinicians to make treatment decisions, including acute renal failure, prior to an urgent surgery, during life-threatening bleeding, a stroke, suspected accumulation of a drug, and when determining potential drug-drug interactions [5]. Coagulation monitoring can aid in clinical decisions, and clinical pharmacists can make anticoagulant recommendations to doctors according to monitoring results.

We can monitor the international normalized ratio (INR) to assess the effect and safety of warfarin. However, there are no specific monitoring indicators for rivaroxaban. Prothrombin time (PT) clotting times are significantly influenced by the thromboplastin used in varying PT reagents. The activated partial thromboplastin time (aPTT) has poor sensitivity and specificity and lacks an optimal dose-response relationship for monitoring rivaroxaban. Several studies [6–8] have shown that there is a linear relationship between anti-factor Xa activity and the concentration of rivaroxaban, and Kozue et al [9] indicated that measurement of anti-factor Xa activity might be useful for assessing the pharmacodynamics of high-risk patients. However, anti-factor Xa activity is not widely used in clinical monitoring. In addition, there are differences in coagulation function between Chinese and Caucasian populations. There are longer aPTT as well as lower protein C and S levels in Chinese individuals than in Caucasian individuals [10]. Individuals of East Asian origin (Chinese and Japanese) has been reported to have a significantly lower risk of venous thromboembolism [11, 12]. However, there are no available studies on rivaroxaban monitoring in Chinese patients. Moreover, there is no specific, approved treatment in China for if a patient bleeds after taking rivaroxaban.

### Aim of the study

The aim of our study was to determine rivaroxaban concentrations in real-world Chinese patients with deep vein thrombosis (DVT) by an ultra-performance liquid chromatography-tandem mass spectrometry (UPLC-MS/MS) method to initially explore the correlation of the plasma concentration range and PT, aPTT, and anti-factor Xa activities and to determine a method for clinical monitoring of rivaroxaban.

### Ethics approval

The study protocol was approved by the Ethics Committee of Peking Union Medical College Hospital (ZS-1359). All subjects signed informed consent forms before the trial.

## Methods

### Chemicals and reagents

**Fig. 1:**
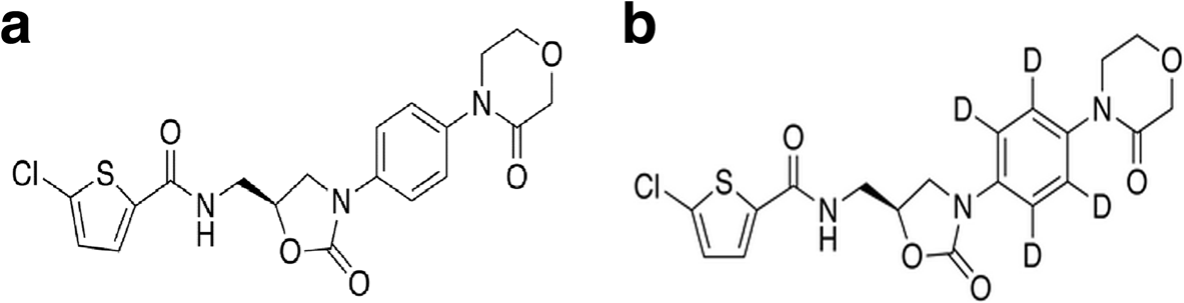
**a** Rivaroxaban chemical structure; **b** Rivaroxaban-d4 chemical structure

Rivaroxaban was provided by Bayer HealthCare AG (Wuppertal, Germany) (Fig. 1a). Rivaroxaban-d4, used as an internal standard (IS), was purchased from Toronto Research Chemicals (Canada) (Fig. 1b). Methanol and acetonitrile of HPLC grade were purchased from Thermo Fisher (MA, USA). LCMS-grade formic acid and dimethyl sulfoxide (DMSO) of analytical grade were purchased from JK Chemical (Beijing, China) and Sigma–Aldrich (France), respectively. Water was purified with a Milli-Q system (Millipore Waters, Darmstadt, Germany).

### Calibrator and quality control sample preparation

The powdered compound of rivaroxaban and rivaroxaban-d4 were dissolved in DMSO to prepare stock solutions at 100 μg/mL and then the stock solutions stored at − 20 °C. Further dilutions were made in methanol to obtain series of intermediate and final working solutions. Then the appropriate amount of working solutions were added in blank human plasma to get the calibration curve with concentrations 0.5, 1, 2, 10, 20, 100, 200, and 400 ng/mL and quality control (QC) samples with concentrations 1.5, 15, and 300 ng/mL.

### Instrument and analytical conditions

An Acquity UPLC system (WATERS, Milford, MA, USA) with an autosampler temperature of 10 °C and Acquity UPLC BEH C18 column (2.1 mm × 50 mm, 1.7 μm particle size; Waters, Milford, MA, USA) was applied to determine the samples in this study. The mobile phase consisted of acetonitrile (A) and ultrapure water (B), and the gradient programme of the mobile phase was as follows: 32% A at 0–1.5 min; 32–90% A at 1.5–1.51 min; 90–32% A at 2.5–2.51 min; and 32% A at 2.51–3 min. The flow rate and the column temperature were set at 0.4 mL/min and 35 °C, respectively.

The analytes were detected in the Acquity tandem quadrupole detector (Waters Xevo TQ-S, Milford, MA, USA) with positive electrospray ionization (ESI^+^) and multiple-reaction monitoring (MRM) mode. And the mass transitions were m/z 437 → 145.0 and m/z 440.1 → 145.0 for rivaroxaban and rivaroxaban-d4, respectively. The operating parameters were as follows: cone voltage, 35 V; collision voltage, 30 V; collision gas flow, 0.16 mL/min; nebulizer gas pressure, 7.0 bar; and desolvation temperature, 500 °C. The retention times were 1.03 min for both rivaroxaban and rivaroxaban-d4.

### Sample pretreatment

Sample preparation was performed by protein precipitation with acetonitrile. A 50 μL aliquot of plasma sample and 150 μL of acetonitrile containing the IS at a concentration of 10 ng/mL were mixed and vortexed for 2 min, then the mixture was centrifuged at 13000 r/min for 10 min at 25 °C.The supernatant was dried under nitrogen at normal temperature, redissolved with 32% acetonitrile and 68% ultrapure water containing 0.2% formic acid and vortexed. After filtering through a 0.22 μm micro-membrane filter, the supernatant was transferred to an autosampler vial and 10 μL was injected into the UPLC system automatically.

### Study design and patients

The study population consisted of adult subjects with deep venous thrombosis (DVT) from Peking Union Medical College Hospital. Eligible subjects were those treated with rivaroxaban and aged 18 or older. Subjects were ineligible if they had severe damage to liver and kidney function, severe cardiopulmonary insufficiency, or they combined other anticoagulants, such as CYP450 3A4 and strong P-glycoprotein inhibitors.

The clinician conducted drug administration based on the patients’ condition. There were some patients who did not have very severe clots or bleeding after taking 20 mg rivaroxaban. Clinicians typically consider giving these patients 10 mg bid or 15 mg qd rivaroxaban. Rivaroxaban (Bayer HealthCare AG, Wuppertal, Germany) was taken with food. When concentrations of rivaroxaban reached a steady state (day seven or later), blood samples were taken 2 h after administration and before the next dose. To ensure patient adherence, we sent text messages to patients to remind them to take the medication and asked patients to fill out medication record forms. Patients continued to take rivaroxaban for at least 3 months. All samples were centrifuged for 10 min at 3000×g, and the plasma was then stored at − 80 °C until analysis.

### Sample analysis

Rivaroxaban plasma concentrations were determined by UPLC-MS/MS. PT, aPTT and anti-factor Xa activity were determined at the same time in the clinical laboratory of Peking Union Medical College Hospital.

Statistical analyses were performed using SPSS software (SPSS for Windows, version 20.0, IBM Corp, Armonk, NY, USA). The arithmetic mean was calculated, and the results are presented as the mean ± standard deviation (SD). The association between PT, aPTT, anti-factor Xa activity and rivaroxaban plasma concentrations by UPLC-MS/MS was determined by Spearman correlation.

## Results

### Method validation

#### Selectivity

**Fig. 2:**
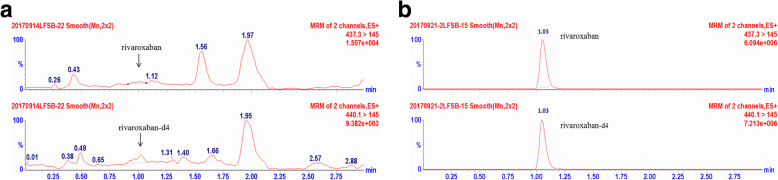
The MRM mass chromatograms of rivaroxaban and d4-rivaroxaban: **a** blank plasma; **b** human plasma with 0.5 ng/mL rivaroxaban and 10 ng/mL internal standard

Six lots different blank plasma were determined to analyze selectivity. The retention time (t_*R*_) was 1.03 min for rivaroxaban and IS. The analysis showed no endogenous peaks at the same time, since the responses of endogenous peaks were lower 20% of lower limit of quantification (LLOQ) (Fig. 2).

#### Accuracy and precision

Six samples for each concentration of QC samples (1.5, 15 and 300 ng/mL) were processed and analysed to obtain intra-run precision and accuracy. Then three different sequences were measured successively to obtain inter-run precision and accuracy. The ratio between the measured concentration and the nominal concentration multiplied by 100% was used as the accuracy. The relative standard deviation indicates precision. Intra- and inter-day imprecision and accuracy outcomes of QC samples are shown in Table 1 and were all below ±15%. This method was then determined to be accurate and precise.

**Table 1:** Accuracy, precision, matrix effect and extraction recovery of rivaroxaban concentrations in human plasma

Theoretical concentration (ng/mL)	Intra-run accuracy and precision		Inter-run accuracy and precision		Matrix effect (%)		Extraction recovery (%)	
	Accuracy (%)	Precision- RSD (%)	Accuracy (%)	Precision- RSD (%)	mean ± SD	RSD	mean ± SD	RSD
0.5 (LLOQ)	103.57	14.05	96.68	14.95	–	–	–	–
1.5	88.02	1.80	88.55	2.47	109.78 ± 2.33	2.12	75.18 ± 2.14	2.84
15.0	94.45	0.69	93.34	4.21	113.81 ± 0.67	0.59	79.62 ± 2.54	3.20
300.0	99.12	4.60	99.90	4.13	106.44 ± 0.92	0.86	87.53 ± 3.31	3.78

*RSD* Relative standard deviation, *SD* Standard deviation

#### Linearity

A calibration curve was established by plotting the peak area ratios of rivaroxaban to the IS (Y-axis) versus the nominal concentration of rivaroxaban (X-axis) through weighted least-squares linear regression analysis with a weighting factor of 1/x^2^. The linear regression equation was the mean of three batches discussed in the section titled “Accuracy and precision”, and the equation was y = 0.0047x-0.0119 for rivaroxaban with a correlation coefficient *r*^2^ = 0.996. The linear range was 0.5 to 400 ng/mL, and the accuracy of the LLOQ (0.5 ng/mL) was 80 ~ 120% with the precision≤20%.

#### Matrix effect and extraction recovery

The matrix effect was assessed six times by comparing the concentrations obtained with three solutions at 1.5, 15 and 300 ng/mL in blank plasma extracts with those of standard rivaroxaban solutions at the same concentrations. The extraction recovery was determined six times by comparing three levels of samples (1.5, 15 and 300 ng/mL) with reference solutions containing blank plasma extracts spiked with rivaroxaban at the same concentrations. The results are shown in Table 1 and remained stable over the linear range.

#### Stability

Three concentrations (1.5, 15 and 300 ng/mL) of rivaroxaban in plasma samples were assessed six times respectively, and then these plasma samples were stored at room temperature (25 °C) up to 24 h, at − 30 °C up to 3 months, in an autosampler at 10 °C up to 48 h and repeatedly frozen and thawed 3 times. Stability was defined as the ratio of each concentration to the concentration of the first day. The results are presented as the mean ± SD (Table 2). Rivaroxaban was stable under all tested conditions since the difference of average measured concentrations and theoretical concentrations was within ±15%.

**Table 2:** Rivaroxaban stability in spiked samples

Theoretical concentrations (ng/mL)	Room temperature (25 °C) up to 24 h	−30 °C up to 3 months	In autosampler at 10 °C up to 48 h	Frozen and thawed 3 times
1.5	92.23 ± 4.84	89.67 ± 1.47	90.65 ± 2.00	95.08 ± 1.04
15.0	85.45 ± 1.05	96.08 ± 1.38	97.78 ± 2.27	104.35 ± 1.03
300.0	95.45 ± 1.26	102.68 ± 2.89	107.23 ± 2.03	110.92 ± 1.62

### Patient concentrations

#### Subjects

Of the 44 patients enrolled in the early study, 5 were eliminated because of no follow-up; 73 plasma samples from 39 subjects were included in these analyses. Based on the condition, these patients took rivaroxaban 15 mg twice daily (BID, *n* = 3), 10 mg twice daily (BID, *n* = 9), 20 mg once daily (QD, *n* = 8), 15 mg once daily (QD, *n* = 7), or 10 mg once daily (QD, *n* = 12). The groups were well matched with respect to demographic characteristics (Table 3). The mean age of the subjects was 56.9 years. Minor between-group differences in BMI, CrCl, ALT and Alb were not statistically significant.

**Table 3:** Demographic characteristics of subjects enrolled in the study

		15 mg BID (*n* = 3)	10 mg BID (*n* = 9)	20 mg QD (*n* = 8)	15 mg QD (*n* = 7)	10 mg QD (*n* = 12)	Total (*n* = 39)	*P*
Demographic characteristics	Age	50.0 ± 3.0	56.4 ± 13.3	49.1 ± 17.6	60.9 ± 10.5	63.4 ± 14.6	56.9 ± 14.8	0.147
	BMI (kg/m^2^)	26.1 ± 2.3	23.7 ± 2.5	23.2 ± 3.8	26.6 ± 4.4	22.9 ± 2.4	23.9 ± 3.3	0.126
	CrCl (mL•min^−1^)	110.4 ± 31.5	100.6 ± 27.5	91.9 ± 35.1	84.4 ± 20.1	76.6 ± 34.2	89.6 ± 31.5	0.334
	ALT (U•L^−1^)	18.7 ± 3.5	17.0 ± 8.2	30.4 ± 12.8	23.8 ± 16.4	18.7 ± 12.1	22.1 ± 12.5	0.092
	Alb (g•L^−1^)	43.7 ± 6.7	39.9 ± 5.1	42.2 ± 6.4	39.7 ± 5.9	41.8 ± 4.0	41.3 ± 5.2	0.694

#### Plasma concentrations

The steady-state trough concentrations in patients with DVT that took 15 mg rivaroxaban BID, 10 mg BID, 20 mg QD, 15 mg QD, and 10 mg QD were 168.5 ng•mL^− 1^ (95% CI, 162.5 to 499.5 ng•mL^− 1^), 74.2 ng•mL^− 1^ (95% CI, 44.7 to 103.6 ng•mL^− 1^), 25.7 ng•mL^− 1^ (95% CI, 10.0 to 42.3 ng•mL^− 1^), 24.5 ng•mL^− 1^ (95% CI, 11.4 to 37.4 ng•mL^− 1^) and 15.4 ng•mL^− 1^ (95% CI, 7.6 to 23.2 ng•mL^− 1^), respectively. The steady-state peak concentrations were 280.1 ng•mL^− 1^ (95% CI, 99.3 to 659.4 ng•mL^− 1^), 271.4 ng•mL^− 1^ (95% CI, 109.0 to 361.7 ng•mL^− 1^), 306.8 ng•mL^− 1^ (95% CI, 240.3 to 376.6 ng•mL^− 1^), 306.4 ng•mL^− 1^ (95% CI, 222.4 to 390.3 ng•mL^− 1^) and 229.2 ng•mL^− 1^ (95% CI, 170.0 to 288.4 ng•mL^− 1^) for the abovementioned dosages, respectively. There was a statistically significant difference (*p* = 0.008) in the trough concentration between the two dosage groups of 10 mg BID and 20 mg QD, but there was no statistically significant difference (*p* = 0.521) in the peak concentration. The plasma concentration in patients who took 10 mg rivaroxaban BID was more stable than that in patients who took 20 mg rivaroxaban QD.

#### Pharmacokinetic and pharmacodynamic correlation

**Fig. 3:**
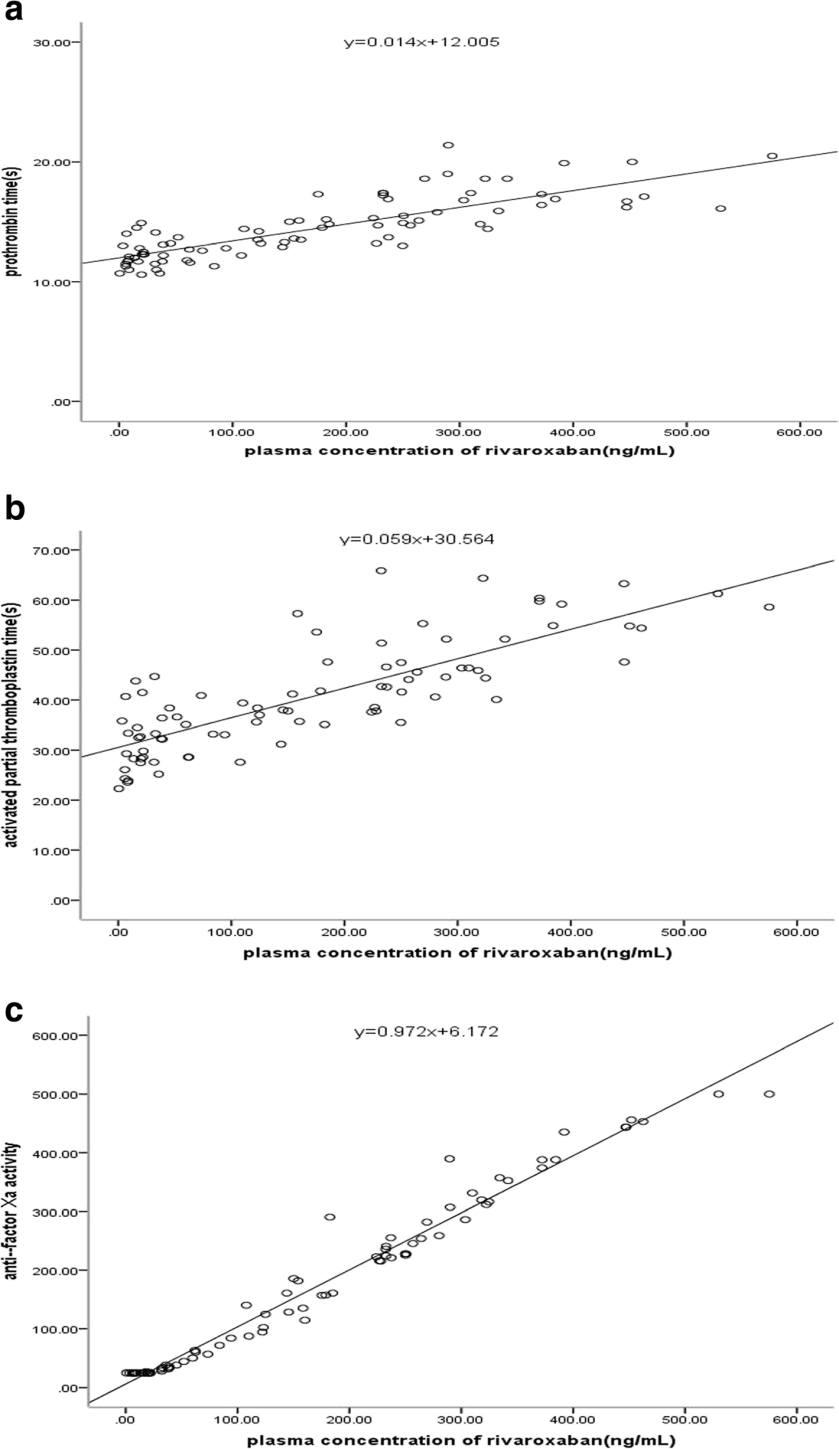
Correlation between plasma concentration of rivaroxaban and PT (**a**), aPTT (**b**) or anti-factor Xa activity (**c**)

Anti-factor Xa activity, PT and aPTT were correlated with the plasma concentration of rivaroxaban (*r* = 0.985, *r* = 0.827 and *r* = 0.807, respectively) (Fig. 3). There was a linear relationship between concentration and anti-factor Xa activity.

## Discussion

On account of predictable anticoagulant effects and relatively low bleeding risk or few drug interactions, new oral anticoagulants have become more widely available for clinical use. The European Heart Rhythm Association (EHRA) guidelines [13] recommend clinical assessment and noncoagulation monitoring every 1 ~ 6 months for patients taking DOACs but do not recommend any monitoring of coagulation assays. However, clinical practices have shown that clinicians need laboratory monitoring to help them make clinical decisions.

In this study, a UPLC-MS/MS method was developed and validated for rivaroxaban quantification using simple sample preparation and chromatographic conditions. In our study of real-world patients under rivaroxaban treatment, we reached an LLOQ of 0.5 ng•mL^− 1^. Previous studies have shown that rivaroxaban samples were stable at 20 °C, + 4 °C and − 20 °C for up to 24 h, 48 h, 5 days, and 1 and 3 months [14, 15]. We first studied the stability of rivaroxaban samples stored at 25 °C for 24 h, − 30 °C for 3 months and in an autosampler at 10 °C for up to 48 h to ensure the stability of rivaroxaban throughout the experiment. Our method was shown to be rapid, specific, reliable and suitable for the determination of rivaroxaban in plasma. This article evaluated the extraction recovery rather than the method recovery, exhibiting an average extraction recovery of approximately 80%. It is possible that the tube used in drying the supernatant under nitrogen had adsorbed some of the rivaroxaban, but the RSD of the three levels of extraction recoveries was 7.74%. Thus, the recovery of this method remained stable over the linear range. We evaluated the relevance of different coagulation assays for determining the rivaroxaban concentration and effect by comparing them with the results of the LC-MS/MS method. PT and aPTT were correlated with the plasma concentration of rivaroxaban in the study, but the relationships were not linear, so they cannot be used for assessing the concentration of rivaroxaban. Douxfils et al [16] indicated that PT may provide some quantitative information, even though the sensitivity of the different PT reagents varies importantly. In contrast, the relationship of anti-factor Xa activity and concentration was linear, so anti-factor Xa activity can be used to estimate concentrations of rivaroxaban. The limitation was that the LLOQ of anti-factor Xa activity was 25 ng•mL^− 1^. At very low concentrations, i.e., ≤25 ng•mL^− 1^, the method is less reliable, and an LC–MS/MS method is still required [14].

The dosage regimens of rivaroxaban are 15 mg BID, 20 mg QD or 10 mg QD in the drug instructions of China. Clinicians often make clinical administration schemes based on the patients’ condition. The real-world patients in the study took either 15 mg rivaroxaban BID, 10 mg BID, 20 mg QD, 15 mg QD or 10 mg QD. Compared with studies of Caucasian patients [17–19], the peak concentration of rivaroxaban was higher in this study (Table 4). The peak concentrations of 10 mg QD and 20 mg QD were 229.2 ng•mL^− 1^ and 306.8 ng•mL^− 1^, respectively, in this study, while they were 124.6 ng•mL^-1^ [17] and 270 ng•mL^− 1^ [18] or 290 ng•mL^-1^ [19], respectively, in Caucasians. The apparent distribution volume of rivaroxaban was approximately 50 ~ 80 L [18–21], which was beyond the total liquid volume, and some rivaroxaban was distributed in tissues or organs. The body mass index (BMI) in previous studies was 27.6–31.6 kg/m^2^ [17, 21], and the maximum BMI of Chinese patients in this study was 26.6 kg/m^2^. However, Kubitza’s study [22] claimed that body weight did not alter rivaroxaban pharmacokinetics. Many factors can affect the rivaroxaban concentration, such as adherence, renal function, co-medication and so on. The sample size of existing research is small; thus, more studies are needed to identify the reasons for the difference.

**Table 4:** Published studies on the pharmacokinetics of rivaroxaban in patients (mean)

Patients	Demographic information	Age	Dosage	C_max_ (ng•mL^−1^)	C_trough_ (ng•mL^− 1^)	CL/F (L•h^− 1^)	Vd (L)
Undergoing total hip replacement [17]	Caucasian	27 ~ 93	10 mg qd	124.6^a^	9.1^a^	7.3	49.1
Deep venous thrombosis [18]	Caucasian	18 ~ 91	20 mg qd	270	25.5	5.67	54.4
Non-valvular atrial fibrillation [19]	Caucasian	51 ~ 92	20 mg qd	290	32	6.1	79.7

^a^median; −, not published

Fox et al. [23] stated that the once daily and twice daily dosing had similar therapeutic effects, and the former had a lower risk of bleeding; therefore, the dosage regimen of rivaroxaban was once daily. In this study, performed on real-life patients with DVT treated with rivaroxaban according to current clinical routines, the trough concentration of 10 mg BID was higher than that of 20 mg QD, and there was no significant difference in the peak concentration. In terms of pharmacokinetics, 10 mg BID rivaroxaban had an advantage over 20 mg QD rivaroxaban because the concentration fluctuated less. We obtained blood samples 2 h after administration and before the next dose. We could not guarantee that every patient did not miss their medication, but we took certain measures, such as sending text messages to patients to remind them to take the medication and asking patients to fill out medication record forms. In addition, the trough concentration was lower in Chinese patients in this study than in Caucasian patients [19], so a dosage of 10 mg rivaroxaban BID may have better efficacy than 20 mg QD. There were significant individual differences in the plasma rivaroxaban concentration, which increased the risk of clinical use of rivaroxaban. Coagulation monitoring is even more necessary for patients with acute renal failure, prior to urgent surgery, during life-threatening bleeding, during a stroke, during overdose and in the suspected accumulation of a drug.

Considering that this was not a clinical trial for a new drug and that there have been similar studies before, we did not perform “incurred sample reanalysis”. We analysed the results from 39 patients. This sample size was too small and was not enough to represent all Chinese patients with DVT. However, the above study was only the early results of a comprehensive study, and additional patients are still enrolled for further research.

## Conclusion

The UPLC-MS/MS method met the requirements of FDA guidelines for biological analysis methods and was rapid, accurate, sensitive and repeatable to determine the concentration of rivaroxaban in Chinese plasma. The LLOQ of 0.5 ng/mL could cover the minimum clinical concentration and the calibration range was 0.5 ~ 400.0 ng/mL. The error of inter- and intra-day accuracy and precision was less than ±15% and the stability of this method met the requirements. This method was successfully applied to plasma samples of 39 Chinese patients, and the plasma concentration range of rivaroxaban was obtained. Moreover, there is a basis for further anticoagulation monitoring research, and anticoagulant clinical pharmacists may provide recommendations for the clinical application of rivaroxaban to promote medication safety in the future.

## Data Availability

The data used in the current study can be accessed by request via the corresponding author.

## Abbreviations

FXa: Factor Xa
VKA: Vitamin K antagonist
INR: International normalized ratio
PT: Prothrombin time
aPTT: Activated partial thromboplastin time
DVT: Deep vein thrombosis
UPLC-MS/MS: Ultra-performance liquid chromatography-tandem mass spectrometry
QC: Quality control
ESI: Electrospray ionization
MRM: Multiple-reaction monitoring
SD: Standard deviation
LLOQ: Lower limit of quantification
EHRA: European Heart Rhythm Association
